# Biodiscoveries within the Australian plant genus *Eremophila* based on international and interdisciplinary collaboration: results and perspectives on outstanding ethical dilemmas

**DOI:** 10.1111/tpj.15866

**Published:** 2022-07-23

**Authors:** Susan J. Semple, Dan Staerk, Bevan J. Buirchell, Rachael M. Fowler, Oliver Gericke, Louise Kjaerulff, Yong Zhao, Hans Albert Pedersen, Malene J. Petersen, Line Fentz Rasmussen, Emilie Kold Bredahl, Gustav Blichfeldt Pedersen, Laura Mikél McNair, Chi P. Ndi, Nikolaj Lervad Hansen, Allison M. Heskes, Michael J. Bayly, Claus J. Loland, Nanna Heinz, Birger Lindberg Møller

**Affiliations:** ^1^ Quality Use of Medicines and Pharmacy Research Centre, Clinical and Health Sciences University of South Australia Adelaide 5000 Australia; ^2^ Department of Drug Design and Pharmacology, Faculty of Health and Medical Sciences University of Copenhagen DK‐2100 Copenhagen Denmark; ^3^ Wise Owl Consulting Como Western Australia 6152 Australia; ^4^ School of BioSciences The University of Melbourne Parkville Victoria 3010 Australia; ^5^ Plant Biochemistry Laboratory, Department of Plant and Environmental Sciences University of Copenhagen DK‐1871 Frederiksberg C Denmark; ^6^ Department of Neuroscience, Faculty of Health and Medical Sciences University of Copenhagen DK‐2100 Copenhagen Denmark; ^7^ Present address: Carlsberg Research Laboratory J.C. Jacobsens Gade 4 DK‐1799 Copenhagen Valby Denmark.

**Keywords:** *Eremophila*, *Scrophulariaceae*, chemo‐evolutionary framework, molecular networks, *cisoid*‐diterpenoids, serrulatane, Australia's First Peoples, traditional medicines, benefit sharing, Nagoya Protocol

## Abstract

In a cross‐continental research initiative, including researchers working in Australia and Denmark, and based on joint external funding by a 3‐year grant from the Novo Nordisk Foundation, we have used DNA sequencing, extensive chemical profiling and molecular networking analyses across the entire *Eremophila* genus to provide new knowledge on the presence of natural products and their bioactivities using polypharmocological screens. Sesquiterpenoids, diterpenoids and dimers of branched‐chain fatty acids with previously unknown chemical structures were identified. The collection of plant material from the *Eremophila* genus was carried out according to a ‘bioprospecting agreement’ with the Government of Western Australia. We recognize that several *Eremophila* species hold immense cultural significance to Australia's First Peoples. In spite of our best intentions to ensure that new knowledge gained about the genus *Eremophila* and any potential future benefits are shared in an equitable manner, in accordance with the Nagoya Protocol, we encounter serious dilemmas and potential conflicts in making benefit sharing with Australia's First Peoples a reality.

## Significance Statement

Eremophilas are culturally important plants for many of Australia's First Peoples, and several species are recognized as important sources of traditional medicines, as also supported by a chemo‐evolutionary study across the entire genus. In this Perspectives review article, we outline dilemmas in achieving benefit sharing with Australia's First Peoples.

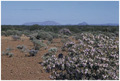

## INTRODUCTION

Eremophilas are culturally important plants for many of Australia's First Peoples, and several species are recognized as important sources of traditional medicines. Previous investigations of the chemical nature of their bioactive constituents were limited to a small number of species (Barr et al., 1993; Chinnock, 2007; Ghisalberti, 1994; Ghisalberti et al., 1975; Singab et al., 2013). With support from the Novo Nordisk Foundation, we established an interdisciplinary, cross‐continental, synergy program, ‘Desert‐loving therapeutics’. Our aims were: (i) to understand the relationships within and beyond the genus *Eremophila* at the DNA and phytochemical levels; (ii) to identify natural products with unique chemical structures; (iii) to ascertain the potential of such natural products as lead compounds for the development of new pharmaceuticals; and (iv) to elucidate the biosynthetic routes for a selected set of natural products as the basis for bioproduction in heterologous hosts. In this Perspectives review article, we provide a short account of the results obtained and outline the research set‐up that made the study possible, including obtaining a ‘bioprospecting agreement’ in Western Australia and the challenges of ensuring that any potential future benefits developed from *Eremophila* can be shared in an equitable manner, in accordance with the Nagoya Protocol. It is important to recognize that many *Eremophila* species hold immense cultural significance to Australia's First Peoples, the Aboriginal Peoples.

## RESULTS

### Natural science

**Figure 1:**
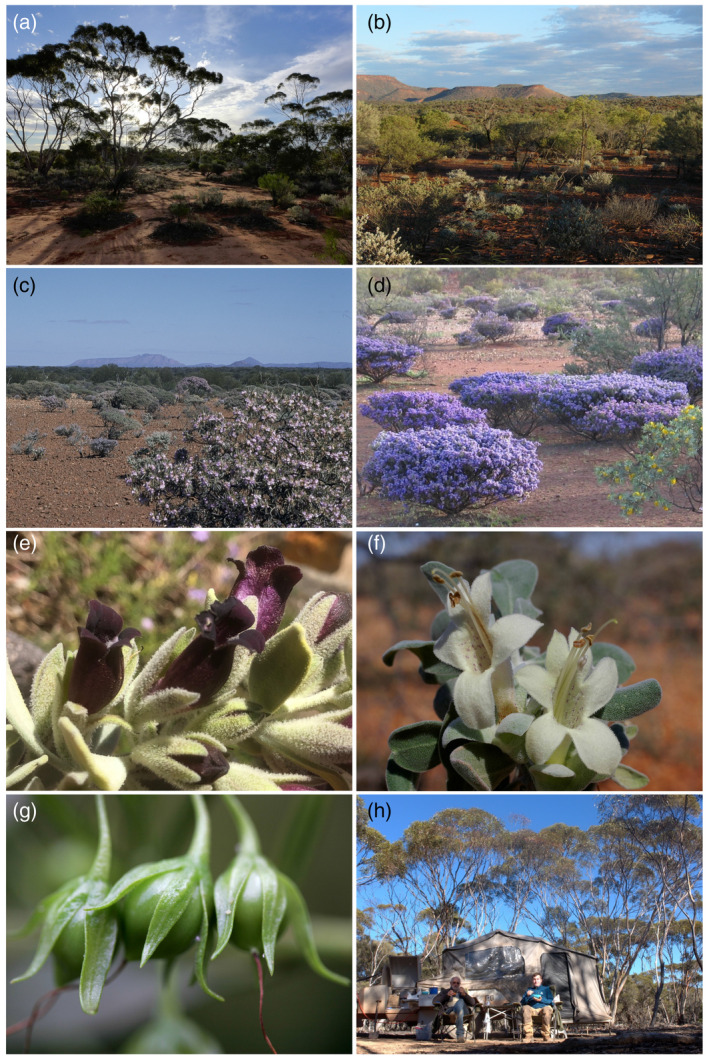
Plant species belonging to the genus *Eremophila* are some of the attractive plants found in the solitudes of the interior arid regions of Western Australia. (a) Landscape east of Kalgoorlie. (b) Landscape north of Gascoyne Junction. (c) Mount Gascoyne, east of Gascoyne Junction. (d) Landscape near Mount Augustus. (e and f) *Eremophila muelleriana* and *Eremophila forrestii*, respectively, demonstrating the diversity of flower pigmentation and the presence of terpenoid‐producing glands on the leaves. (g) *Eremophila* species produce fleshy fruits that enclose between two and 12 seeds. (h) Bevan Buirchell and Oliver Gericke outback camping during a collection, with their trips totaling 83 000 km over the 3‐year program. Photos: Bevan Buirchell and Birger Lindberg Møller. [Colour figure can be viewed at wileyonlinelibrary.com]

**Figure 2:**
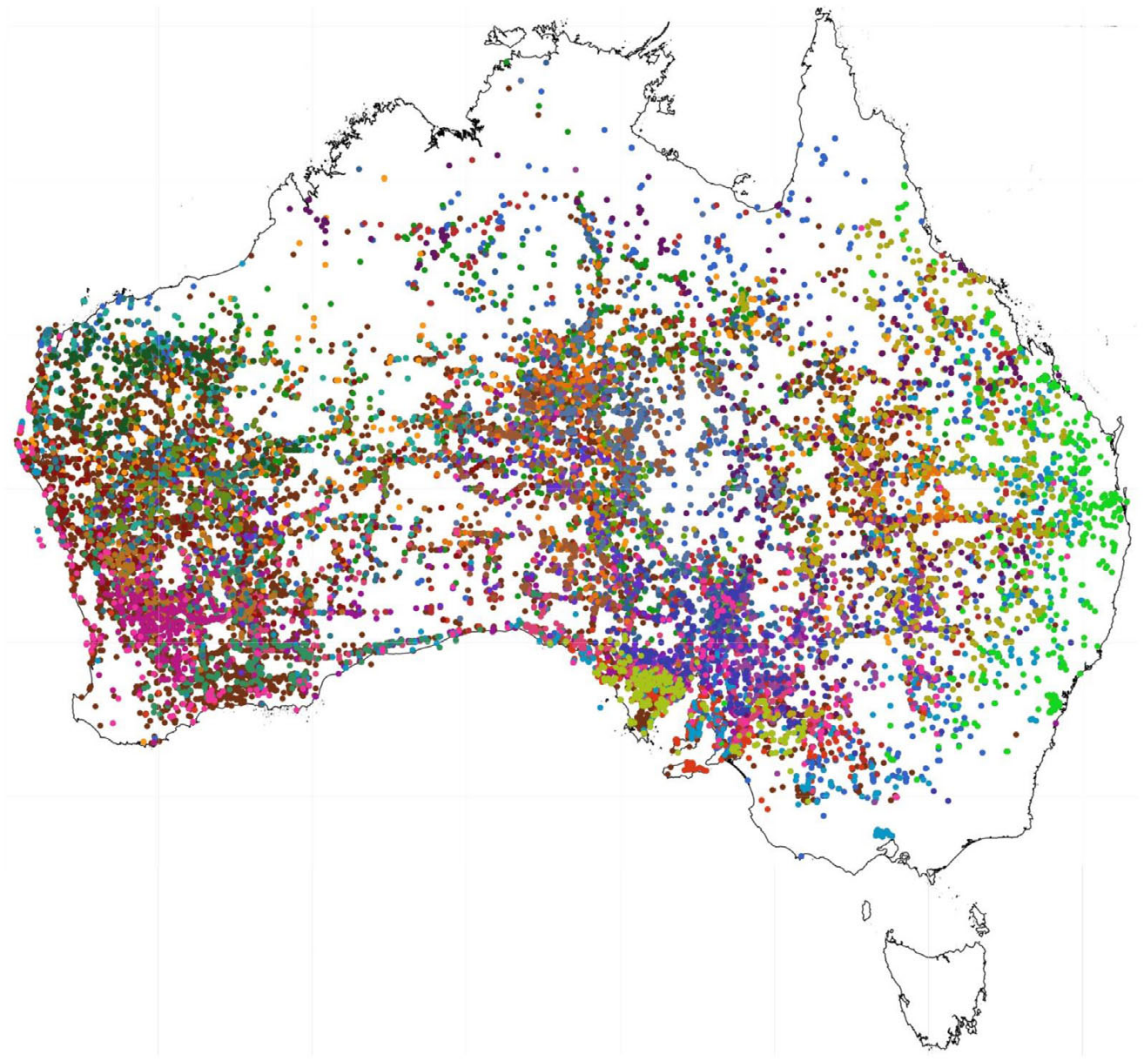
Distribution of the genus *Eremophila* in Australia based on specimen records (Australasian Virtual Herbarium, 2022) using a specific color code for each species shown (Atlas of Living Australia, 2022). [Colour figure can be viewed at wileyonlinelibrary.com]

*Eremophila* R.Br. (Scrophulariaceae) is a large and diverse genus of plants endemic to mainland Australia (Figure 1). As the name of the genus suggests (from the Greek: eremos = desert; philos = loving, i.e. desert‐loving), *Eremophila* species are concentrated in arid Australia, including a notable diversity hotspot in the remote regions of Western Australia (Figure 2). *Eremophila* is the largest genus within the plant tribe Myoporeae (Fowler et al., 2020, 2021). The aim of our study was to obtain new knowledge on the bioactivities of the natural products present across the entire genus of *Eremophila* using DNA sequencing and extensive chemical profiling (Gericke et al., 2021). The summarized results presented here were obtained by a highly multidisciplinary cross‐continental team, with research groups located in Adelaide, Perth, Melbourne and Copenhagen. The main roles of the principal investigator (PI) and individual co‐PIs are outlined in Box 1. The 3‐year research initiative was funded by the Novo Nordisk Foundation Interdisciplinary Synergy program.

Box 1 **Bevan Buirchell (ORCID: 0000‐0001‐8247‐3570), Wise Owl Consulting, Como, Western Australia:** Bevan Buirchell is an Australian biological scientist with a cross disciplinary expertise in botany, biochemistry and plant genetics with a long career as a Plant Breeder with the Government of Western Australia and a special focus on lupin breeding (Berger et al., 2013; Yang et al., 2013). Bevan Buirchell is an expert on botanical classification of *Eremophila* species in Western Australia and has unique knowledge of their specific often remote growth sites. He is author of “A Field Guide of the *Eremophilas* of Western Australia” (Brown and Buirchell, 2011).

**Michael J. Bayly (ORCID: 0000‐0001‐6836‐5493), Rachael Fowler (ORCID: 0000‐0002‐8953‐7036), School of Biosciences, University of Melbourne, Australia:** Mike Bayly, Rachael Fowler and their research group use DNA data to study the evolution, diversity and classification of the Australian flora including large and iconic plant groups such as eucalypts (Schuster et al., 2018), ferns (Ohlsen et al., 2020) and bryophytes (Meagher and Bayly, 2014). Their team uses high‐throughput DNA sequencing for assembly of whole chloroplast genomes (Fahey et al., 2020, Fowler et al., 2020), nuclear ribosomal arrays (Fowler et al., 2020) and genome‐wide nuclear markers (Fahey et al., 2020). To expand their research on the Australian flora, a long‐term collaboration was established with Bevan Buirchell for collection of native species from a range of genera including *Eremophila*. A collection of leaf samples from 346 plant specimens representing approximately 88% of the species in the tribe were previously established, the large majority from Western Australia (Fowler et al., 2021). Most of the specimens used for phylogenetic analyses (Fowler et al., 2020, 2021) were collected between 2013–2016 under Australian state and territory scientific flora licensing. Phylogenetic data generated based on DNA sequences from whole chloroplast genomes (Fowler et al., 2020) and nuclear ribosomal DNA (Fowler et al., 2021) showed that *Eremophila* is not a single evolutionary lineage and that the genera *Bontia, Calamphoreus, Diocirea, Glycocystis, Myoporum* and *Pentacoelium* are nested within it. This knowledge has led to the re‐classification of two genera (*Calamphoreus* and *Diocirea*), now recognized as *Eremophila* (Bayly et al., 2020; Fowler et al., 2021), and further generic level taxonomic change is imminent. The sequencing also demonstrated that some currently‐recognized *Eremophila* species should be treated as a number of distinct species, whose taxonomic limits need assessment (Fowler et al., 2021). A system was therefore in full operation from the start of the Novo Nordisk Foundation research initiative to collect *Eremophila* samples based on the “Bioprospecting Agreement”, secure proper storage and handling and subsequent courier transport to the laboratories in Adelaide, Melbourne and Copenhagen.

**Dan Stærk (ORCID: 0000‐0003‐0074‐298X), Louise Kjærulff (ORCID: 0000‐0001‐6274‐4265); Yong Zhao (ORCID: 0000‐0002‐0423‐5232); Hans Albert Pedersen (ORCID: 0000‐0003‐2289‐7384), Malene J. Petersen (ORCID: 0000‐0002‐0531‐8531); Line Fentz Rasmussen (ORCID: 0000‐0003‐0842‐6770); Emilie Kold Bredahl (ORCID: 000‐0002‐3709‐3779), Department of Drug Design and Pharmacology, University of Copenhagen**: Dan Stærk is heading the Natural Products Research group and the Copenhagen Small‐Molecule NMR Center. He and his research group have discovered and structurally characterized a wealth of small‐molecule natural products from sources like medicinal plants, insects, marine organisms, microorganisms and food by‐products (Liu et al., 2016; McNair et al., 2018; Lima et al., 2019; Gimenes et al., 2020; Malik et al., 2020; Nair et al., 2020; Liang et al., 2021). Many of these have shown great potential as drug leads within disease areas covering type 2 diabetes (T2D), infectious diseases (antibacterial and antifungal compounds), malaria and cancer, the main aim being to foster sustainable drug discovery. A central part of this research is development and application of new state‐of‐the‐art bioanalytical techniques for advanced chemical and pharmacological profiling of complex extracts. This includes high‐resolution polypharmacological inhibition profiling (Zhao et al., 2018, 2019b; Li et al., 2019; Ardalani et al., 2021; Liang et al., 2021) and ligand fishing (Wubshet et al., 2015; Petersen et al., 2019) for identification of bioactive constituents in crude extracts ‐ followed by high‐performance, liquid chromatography, photodiode array detection, high‐resolution mass spectrometry, solid phase extraction and nuclear magnetic resonance spectroscopy (HPLC‐PDA‐HRMS‐SPE‐NMR) analysis for full structural identification of individual constituents (Lima et al., 2017; Zhao et al., 2018). Similarly, bioactivity‐correlated metabolomics and bioaffinity NMR are techniques regularly employed to study bioactive natural products (Liu et al., 2017; Broholm et al., 2019).

**Birger Lindberg Møller (ORCID: 0000‐0002‐3252‐3119), Oliver Gericke (ORCID: 0000‐0002‐8638‐6797), Allison Maree Heskes (ORCID: 0000‐0002‐2732‐5185), Nikolaj Lervad Hansen (ORCID: 0000‐0002‐5938‐16591), Gustav Blichfeldt Pedersen (ORCID: 0000‐0002‐7830‐718X), Plant Biochemistry Laboratory, University of Copenhagen:** Birger Lindberg Møller is a plant biochemist specializing in the biosynthesis and function of plant natural products, metabolomics, metabolic engineering and environmentally benign production of natural products using heterologous hosts (Luo et al., 2017; Knudsen et al., 2018). A special focus has been on pathways in which the coveted steps are catalyzed by membrane bound cytochrome P450 enzymes (Knudsen et al., 2018; Jensen et al., 2021). Within the diterpenoid area, pathways for ingenol‐3‐angelate (Picato^R^ for treatment of actinic keratosis) (Luo et al., 2016), forskolin (cyclic AMP booster for weight loss) (Pateraki et al., 2014, 2017; Forman et al., 2018; Jensen et al., 2021), ginkgolides (Forman et al., 2022) and triptonide (Hansen et al., 2022) have been studied and elucidated based on metabolite profiling, transcriptomics, network analyses and functional expression of the gene candidates in tobacco and yeast (Andersen‐Ranberg et al., 2016). The Plant Biochemistry Laboratory hosts a state‐of‐the‐art metabolomics platform. Structural analysis of isolated intermediates using NMR spectroscopy is being carried out with Dan Staerk (Gericke et al., 2020; Kjaerulff et al., 2020; Pedersen et al., 2020; Bredahl et al., 2022). As Head of a number of major research centers, most recently the VILLUM Center for Plant Plasticity and the Center for Synthetic Biology, Birger Lindberg Møller is an experienced research director.

**Susan Semple (ORCID: 000‐0001‐5988‐3993) and Chi P. Ndi (ORCID: 0000‐0003‐4198‐3237), Quality Use of Medicines and Pharmacy Research Centre, University of South Australia**: Susan Semple's group has a strong background in pharmacy and natural products and has published widely on Australian medicinal plants including *Eremophila*. A specific focus area of the research group has been identification of natural products and derivatives used in treatment of infections caused by multi‐drug resistant bacteria with inhibitors of bacterial efflux pumps being key targets (Ndi et al., 2007; Mon et al., 2015; Biva et al., 2016; Gu et al., 2020).

Susan Semple has also pioneered the establishment of collaborative partnerships in projects driven by Australian Aboriginal communities themselves (Claudie et al., 2012). Working with the Chuulangun Aboriginal Corporation, a variety of bioactive compounds including novel anti‐inflammatory diterpenoids from plants used in traditional medicine were identified (Simpson et al., 2014). These diterpenoids formed the basis of a joint patent application between the Aboriginal Corporation and the University of South Australia. This has been used by the Australian Government organization Intellectual Property (IP) Australia as a case study for Indigenous Peoples’ IP protection (https://www.ipaustralia.gov.au/about‐us/public‐consultations/indigenous‐knowledge‐consultation/chuulangun‐aboriginal‐corporation) and by the Government of the Australian state of Queensland (Queensland Government Biodiscovery Resource Toolkit, 2021) which has recently reformed the state´s *Biodiscovery Act* to introduce protections for First Nations peoples´ traditional knowledge in biodiscovery (The State of Queensland, 2021). This represented a unique starting point for initiation of the research project funded by the Novo Nordisk Foundation where we wanted to adhere to the principles of the Nagoya Protocol around fair and equitable sharing of benefits arising from the utilization of genetic resources and associated traditional knowledge and include Australia's First Peoples, the Aboriginal peoples of Australia, for whom Eremophilas are culturally important plants, as beneficiaries of the project outcomes.

**Claus J. Loland (ORCID: 0000‐0002‐1773‐1446), Laura Mikél McNair (ORCID: 0000‐0002‐5213‐9931), Department of Neuroscience, University of Copenhagen:** Claus Loland is an expert on the molecular pharmacology of membrane transporters with focus on the neurotransmitter:sodium symporter (NSS) class of proteins. The binding of ligands and the molecular mechanisms, which mediate the transport of substrates across the cell membrane are elucidated at atomic level resolution (Billesbølle et al., 2016; Nielsen et al., 2019; Gotfryd et al., 2020; Mortensen and Loland, 2020; Plenge et al., 2021). The binding sites and binding modes for substrates and inhibitor drugs are mapped with special expertise in finding compounds with either orthosteric or allosteric binding properties. Studies on the dopamine transporter (DAT) have particular focus due to its central role in regulating dopamine signaling and as the main target for the binding of illicit drugs such as cocaine and amphetamine (Beuming et al., 2008).

In 2017, the Department of Parks and Wildlife within the Western Australian Government was approached to negotiate and obtain a license agreement to collect *Eremophila* species in Western Australia. The negotiations were carried out in good faith between the Australian authority and Bevan Buirchell and Birger Lindberg Møller in face‐to‐face meetings in Perth. In 2017, the ‘bioprospecting agreement’ was finalized and signed, with great help from Senior Consultant Eva Lessèl from the University of Copenhagen Tech Trans Office. According to the bioprospecting agreement, Bevan Buirchell was permitted to collect representative samples of *Eremophila* species at different stages of plant ontogeny (e.g. young leaves, old leaves, flowers and stem pieces). It was specified how voucher specimens should be processed and deposited at the Western Australian Herbarium (https://www.dpaw.wa.gov.au/plants‐and‐animals/wa‐herbarium) for incorporation into the collection. The identification of new *Eremophila* taxa was to be reported to the Herbarium Curator. *Eremophila* species listed as ‘Declared Rare Flora’ were exempt from any collection. In 2020, the time frame of the bioprospecting agreement was renegotiated and, as a result, extended to 2027 and expanded to include the collection of root samples. The fees paid to the Department of Parks and Wildlife for each sample collected have been used for flora research.

During the 3‐year funded research initiative, and based on the negotiated bioprospecting agreement, Bevan Buirchell headed *Eremophila* expeditions covering more than 80 000 km, mainly across the Western Australian arid zone (Figures 1 and 2). Leaf tissues of more than 250 different *Eremophila* species, subspecies and possibly new species were collected, sometimes revisiting remote sites for additional sampling to obtain sufficient material for a more detailed characterization of the natural products present (Figure 2). The collection of *Eremophila* species was guided by prior and parallel phylogenetic studies to ensure optimal coverage of the phylogenetic and chemical space within the genus (Fowler et al., 2020, 2021). As a result, a unique collection of *Eremophila* species representing approximately 80% of the species in the plant tribe Myoporeae was established. An additional result of the field collection was the collection and identification of six new species of *Eremophila*, although these are yet to be described.

**Figure 3:**
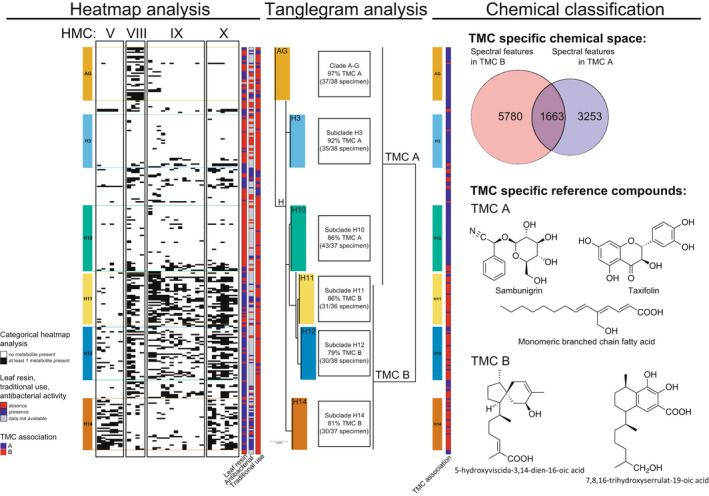
Exploring the plant tribe Myoporeae in a chemo‐evolutionary context. To describe relationships between the evolutionary history and chemical space present in the plant tribe Myoporeae, phylogenetic and chemical information of a diverse set of 291 specimens was generated and analyzed. A heat map analysis was used to cojoin chemical and phylogenetic information. Using molecular networking, 100 major chemical families were isolated and their presence (black) or absence (white) is displayed for each specimen that are given a phylogenetic sequence. In this way, subsets of chemical families sharing an evolutionary signature emerged from the heat map and were assigned to heat‐map metabolic clusters (HMCs). Distinct phylogenetic clades (highlighted in color to the left) associated with certain HMCs (here shown are HMC V, VIII, IX and X), displaying the inherent chemical nature as a chemo‐evolutionary fingerprint for these species. Additional overlap of HMCs (e.g. HMC X) with functional annotations (i.e. presence/absence of leaf resin) led to the hypothesis of a chemistry‐based adaptive evolution in Myoporeae. A tanglegram analysis directly links the Myoporeae phylogeny with clustered metabolite profiles, whereas the profiles revealed a major split of the metabolome into two distinct tanglegram metabolic clusters (TMCs). For each major phylogenetic clade, the percentage and total number of species sharing a similar metabolic background (TMC A or B) is given. A thorough chemical classification of the components found in TMC A (blue) and TMC B (red) was conducted utilizing the chemical annotations provided by the molecular networking approach. Herein, TMC A contains a diverse range of metabolites, whereas TMC B is rich in metabolites from the serrulatane and viscidane diterpenoid class. For all experimental details, see Gericke et al. (2021). [Colour figure can be viewed at wileyonlinelibrary.com]

The genus *Eremophila* is part of tribe Myoporeae and is a significant component of the Australian arid zone flora (Figure 1). Generic limits and relationships with the other genera of tribe Myoporeae were historically uncertain (Kelchner, 2003). A high‐throughput DNA gene sequencing approach based on a prior collection of 346 plant specimens was designed to resolve these phylogenetic relationships (Fowler et al., 2020, 2021). A subset of 291 specimens representing 80% of the species in the tribe was included in the chemo‐evolutionary analysis. Phylogenetic analyses of these data identified eight well‐supported major lineages (Figure 3) (Gericke et al., 2021). The study highlighted a complicated evolution of *Eremophila*, including polyploidy and introgression between species. *Eremophila* was shown to be paraphyletic, with all other genera of the tribe Myoporeae nested within it. Two taxonomic options for addressing the paraphyly of *Eremophila* were presented and discussed (Bayly et al., 2020; Fowler et al., 2021). The DNA‐based phylogeny was developed as rapidly as possible, with intermediate results being made available to other partners in the consortium to optimize the sampling of *Eremophila* species to secure the best possible coverage of phylogenetic diversity in subsequent chemical analyses.

**Figure 4:**
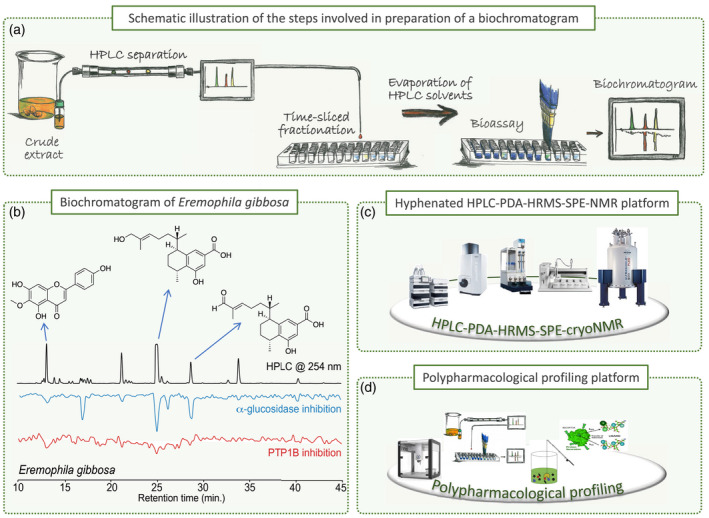
Schematic illustration of the steps in high‐resolution inhibition profiling in the preparation of a biochromatogram. (a) An aliquot of a crude *Eremophila* extract is applied and separated using an analytical‐scale HPLC column and microfractionation of the eluate into 96‐well microplates. After evaporation of the eluate fractions collected, the biological activity of the content of each well is assessed in a bioassay and the results expressed as percentage inhibition are plotted against the retention time of each well to provide a biochromatogram (high‐resolution inhibition profile). Multiple microfractionations and different bioassays are used for polypharmacological profiling. (b) Dual α‐glucosidase/PTP1B biochromatogram of a crude extract of leaves of *Eremophila gibbosa* and identification of selected compounds. (c) Hyphenated HPLC‐PDA‐HRMS‐SPE‐NMR platform used for the accelerated identification of constituents directly from the analytical‐scale HPLC separation of crude extracts. (d) Polypharmacological profiling platform allowing high‐resolution inhibition profiling, ligand fishing, bioactivity‐labeled metabolomics and bioaffinity NMR.

**Figure 5:**
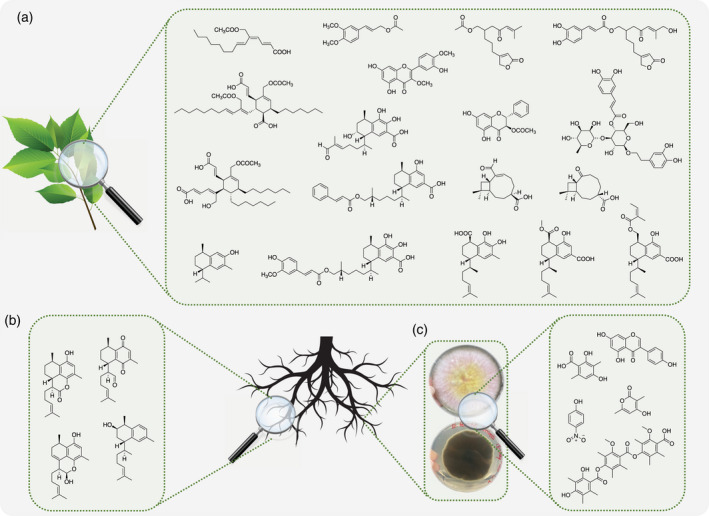
Illustration of the chemical diversity in *Eremophila* spp. (a) Flavonoids, sesquiterpenoids, diterpenoids and branched‐chain fatty acids from leaves of various *Eremophila* spp. (b) Serrulatane diterpenoids isolated from root material. (c) Metabolites isolated from endophytic fungi isolated from roots of *Eremophila* spp.

The chemical space of leaf material from the selected *Eremophila* species and from species in allied genera was initially screened using a hyphenated analytical system based on high‐performance liquid chromatography, photodiode array detection and high‐resolution mass spectrometry (HPLC‐PDA‐HRMS) (Figure 3) (Gericke et al., 2021). The combination of DNA‐based phylogenetics and chemosystematic analyses enabled the identification of patterns of chemical evolution and molecular networks (Figure 3). Clear phylogenetic patterns, with closely related species having similar chemical profiles, were documented in tanglegrams and eight metabolic clusters were identified (Figure 3) (Gericke et al., 2021). Based on these results, eight *Eremophila* species were selected as representative species for in‐depth chemical analysis to provide an overview of the chemical diversity across the genus. These chemical analyses, e.g. HPLC‐PDA‐HRMS combined with semi‐preparative‐scale HPLC and nuclear magnetic resonance (NMR) spectroscopy, identified a large number of sesquiterpenoids and diterpenoids and numerous flavonoids (Figure 4) (Kjaerulff et al., 2020; Petersen et al., 2021; Zhao, Kjaerulff, et al., 2019). A distinct observation from the chemosystematics was the species‐specific presence of a plethora of diterpenoids based on serrulatane‐, viscidane‐ and cembrene‐type scaffolds (Kjaerulff et al., 2020; Petersen et al., 2022; Tahtah et al., 2016; Wubshet et al., 2016). The structures of 98 hitherto undescribed diterpenoids based on these core skeletons were established using 1D and 2D NMR spectroscopy (Figure 5) (Kjaerulff et al., 2020; Petersen et al., 2022; Zhao, Kjaerulff, et al., 2019). Typically, these compounds accumulated in resins localized at the leaf surface and associated with the occurrence of glandular trichomes (Gericke et al., 2020). Structurally unique sesquiterpenoids were also present in *Eremophila*, and were shown to be of the caryophyllane‐type in *Eremophila spathulata* (Bredahl et al., 2022) and of the 2(5*H*)‐furanone‐type in *Eremophila bignoniiflora* (Zhao, Kjaerulff, et al., 2019). Flavonoids and triterpenoids were also detected in several *Eremophila* species. The diterpenoid alkaloid microthecaline A was isolated from *Myoporum insulare*, a species closely related to *Eremophila* (Kjaerulff et al., 2020). Numerous sesquiterpenoids and diterpenoids were found to be bioactive natural products demonstrating activity against multidrug‐resistant bacteria and cancer (Petersen et al., 2021).

**Figure 6:**
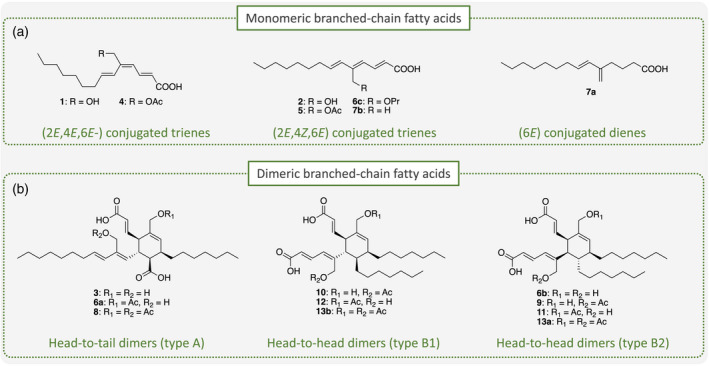
Unusual branched‐chain fatty acids (BCFAs) isolated from leaves of *Eremophila oppositifolia*. (a) Monomeric BCFAs with conjugated double bonds and branching at C5. (b) Dimeric BCFAs formed by Diels–Alder reactions: head‐to‐tail (type A) or head‐to‐head (type B1 and B2, with different stereochemistry). [Colour figure can be viewed at wileyonlinelibrary.com]

Inspired by these results, we continued collecting and investigating more plant material, guided by the results from the molecular networking analysis (Gericke et al., 2021) or polypharmacological screening (Figure 5). Most remarkable was the isolation of numerous highly oxygenated diterpenoids with serrulatane‐type scaffolds (Kjaerulff et al., 2020; Petersen et al., 2022; Tahtah et al., 2016; Wubshet et al., 2016) (Figure 3). Many of these serrulatane‐type diterpenoids showed α‐glucosidase, α‐amylase and/or PTP1B inhibitory activity. Analyses of leaves of *E. spathulata* resulted in the isolation and elucidation of the structure of unique sesquiterpenoids with caryophyllane‐type scaffolds (Bredahl et al., 2022), and leaves of *E. bignoniiflora* revealed a series of 2(5*H*)‐furanone‐type sesquiterpenoids with PTP1B inhibitory activity (Zhao, Kjaerulff, et al., 2019). In previous studies approximately 60 serrulatane diterpenoids have been reported (Ghisalberti, 1994; Ghisalberti et al., 1975; Singab et al., 2013), and the discovery of an additional, hitherto undescribed, 98 serrulatane diterpenoids and 18 sesquiterpenoids in this cross‐disciplinary project – the majority of which demonstrate interesting bioactivity – emphasizes that *Eremophila* is a genus covering a large and as yet unexplored chemical space. A major and truly unexpected surprise came about upon the analysis of *Eremophila oppositifolia*. This species was found to produce a number of PTP1B‐inhibiting branched‐chain fatty acids (BCFAs), i.e. five hitherto unreported monomers and 10 hitherto unreported dimers, originating from head‐to‐tail or head‐to‐head Diels–Alder reactions of monomeric BCFAs (Pedersen et al., 2020) (Figure 6). This class of natural products had not previously been discovered to be produced in plants. Near the end of the program period, the bioprospecting agreement was extended to grant permission to collect root samples. In the root bark of almost all *Eremophila* species investigated, dereplication of HPLC‐PDA‐HRMS profiles revealed the presence of the diterpenoid alkaloid microthecaline A. In addition, a series of 12 hitherto undescribed serrulatane diterpenoids with highly unusual side‐chain modifications and side‐chain cyclizations was discovered in the root bark of *Eremophila longifolia*.

The typical workflow for the above‐mentioned drug discovery projects was as follows: upon the HPLC‐PDA‐HRMS‐ and/or molecular networking‐based detection of new structurally interesting compounds in extracts of one of the *Eremophila* species collected, the crude extract was subjected to polypharmacological screening. If the extract showed sufficiently strong inhibition of one or more of the targets assayed, the eluate from analytical‐scale HPLC was subjected to time‐based micro‐fractionation in one or more 96‐well microplates (Figure 4a). After the enzymatic assaying of each well towards the chosen target(s), the results were plotted as function of each well's retention time from the microfractionation. This provided polypharmacological high‐resolution inhibition profiles or ‘biochromatograms’ showing the *in vitro* bioactivities of the natural product present in the collected fraction (Figure 4d) (Zhao et al., 2018; Zhao, Kongstad, et al., 2019; Pedersen et al., 2020). Among the sesquiterpenoids and diterpenoids isolated, several serrulatane diterpenoids displayed inhibitory activity towards mainly PTP1B and α‐glucosidase, two targets relevant for management of type‐2 diabetes (T2D) (Figure 4) (Petersen et al., 2022; Wubshet et al., 2016; Zhao, Kjaerulff, et al., 2019). In addition, some serrulatanes showed antibacterial (synergistic) activity against various clinically relevant strains and a highly methoxylated flavonoid from *Eremophila galeata*, 5,3′,5′‐trihydroxy‐3,6,7,4′‐tetramethoxyflavone, showed the reversal of resistance to the anticancer drug SN‐38 by the inhibition of the breast cancer resistance protein (Petersen et al., 2022). The discovered branched‐chain fatty acid dimers showed high affinity towards the T2D target PTP1B (Figure 6). Pharmacological profiling of the *Eremophila* and *Myoporum* root samples also revealed a large number of new sesquiterpenoids, diterpenoids and triterpenoids with anti‐diabetic activity (Kjaerulff et al., 2020).

For the many serrulatanes identified in this project, the relative stereochemistry was determined using two‐dimensional nuclear Overhauser effect spectroscopy (2D NOESY). For the determination of the absolute stereochemistry, chiroptical methods are needed. Thus, for selected serrulatanes, electronic circular dichroism (ECD) spectra were acquired, and theoretical ECD spectra were calculated by time‐dependent density‐functional theory calculations. This showed a conserved 1*R*,4*S*,11*S* configuration at C1, C4 and C11, in agreement with previous findings from microthecaline A (Kumar et al., 2018) and in agreement with the common 8,9‐dihydroserrulat‐14‐ene backbone (rapidly aromatized to serrulat‐14‐ene) arising from class‐I terpene synthases with *cisoid* nerylneryl diphosphate as the substrate (Gericke et al., 2020).

From the start of the project period, a fraction of many of the *Eremophila* samples was immediately sent to Susan Semple's group for initial extracts to be prepared and subfractionated for the analysis of extracts and compounds with inhibitory effects against *Enterobacteriaceae*, *Pseudomonas* spp., *Acinetobacter baumannii* and multidrug‐resistant *Staphylococcus aureus* and the efflux pump systems of some of these bacteria (Ohene‐Agyei et al., 2014). Leaf extracts of *E. galeata* showed encouraging results in reversing resistance to the antibiotic norfloxacin in *S. aureus* through the inhibition of the efflux pump NorA. These organisms are classified as ‘critical’ or ‘high priority’ for antimicrobial drug development according to the World Health Organization (Willyard, 2017). In this context it is highly interesting that the data obtained show that some serrulatane diterpenoids may be used to repotentiate existing antibiotics towards multidrug‐resistant bacteria.

A wide range of isolated *Eremophila* compounds were screened in detail as regulators of the activity of the neurotransmitter:sodium symporter (NSS) family of proteins. Of special interest was a set of unique branched‐chain fatty acid dimers isolated from *E. oppositifolia* that possess a pharmacological signature that has not been observed previously for any of these types of transporters. These effects have the potential to provide completely new therapeutic perspectives, such as alleviating the psychotic symptoms of patients with schizophrenia without having the severe side effects of the current antipsychotics. The research to substantiate this function and elucidate the molecular mechanism behind the novel pharmacological signature is being given high priority. From previous experiments, it is known that cocaine and cocaine‐like compounds induce an outward‐facing open conformation of the dopamine transporter (DAT) (Loland et al., 2008, 2012). In contrast, a class of atypical DAT inhibitors bind to an inward‐facing conformation of the DAT and distinguish themselves from cocaine by not eliciting any stimulatory or rewarding response (Abramyan et al., 2017; Slack et al., 2020; Zou et al., 2017). The DAT binding conformation induced by the isolated compounds was investigated using DAT mutants that were biased towards specific conformations. It was discovered that several of the compounds had differentiated effects on these mutants, suggesting that they promote certain DAT conformations. Some of these properties have not previously been reported for DAT ligands. Further experiments must be performed to elucidate the intriguing properties on this novel class of NSS binding compounds.

The chemosystematic screen of the *Eremophila* samples revealed the unique presence of diterpenoids based on serrulatane, viscidane and cembrene‐type scaffolds (Figures 3, 4, 5) (Gericke et al., 2021; Ghisalberti et al., 1975). The biosynthesis of these types of diterpenoids was investigated in three species: *Eremophila denticulata* ssp. *trisulcata*, *Eremophila drummondii* and *Eremophila lucida*. The diterpenoids were found to localize to the leaf surface and were associated with the occurrence of glandular trichomes (Zhao, Kjaerulff, et al., 2019; Gericke et al., 2020; Kjaerulff et al., 2020). Trichome‐enriched transcriptome databases were generated and mined for candidate terpene synthases (TPSs). Four TPSs with diterpene biosynthetic activity were identified: *El*TPS31 and *El*TPS3 from *E. lucida* were found to produce (3*Z*,7*Z*,11*Z*)‐cembratrien‐15‐ol and 5‐hydroxyviscidane, respectively (Gericke et al., 2020). *Ed*TPS22 and *Edt*TPS4 from *E*. *drummondii* and *E*. *denticulata* ssp. *trisulcata* were found to produce 8,9‐dihydroserrulat‐14‐ene, which readily aromatized to serrulat‐14‐ene. In all cases, the identified TPSs used the *cisoid* substrate, nerylneryl diphosphate (NNPP), to form the observed products (Gericke et al., 2020). Subsequently, *cis*‐prenyl transferases (CPTs) capable of making NNPP were identified in each species (Gericke et al., 2020). Previously, *cis*‐precursor‐derived terpenoids have been considered evolutionary novelties of the *Solanum* genus, a distant relative of *Eremophila* (Zi et al., 2014).

A major challenge in this part of the project was engineering the diterpenoid pathways into a production host. In other research projects, we have been highly successful in elucidating the full pathways for structurally complex *transoid‐*diterpenoids by first producing the linear C20 isoprene precursor and core structures in yeast, and then identifying the P450s involved in the oxygenation of the core structures by the co‐expression of putative P450‐encoding candidate genes and characterizing their functionality by metabolite profiling (Andersen‐Ranberg et al., 2016; Pateraki et al., 2017; Gülck et al., 2020; Forman et al., 2022; Hansen et al., 2022). In the case of the *Eremophila* diterpene synthases, their use of the *cisoid* precursor became a major stumbling block. We were unable to successfully express any of the NNPP synthases identified from *Eremophila* species in yeast, preventing any production of *cisoid*‐diterpenoids. The co‐culturing of *Escherichia coli* and yeast was tested as an alternative approach for the bioproduction of *cisoid‐*diterpenoids. In this system, the transformed *E. coli* cells produced the *cisoid*‐diterpenoid scaffolds and the yeast‐expressed P450s would then catalyze their subsequent oxygenations. Despite numerous efforts, the co‐culturing approach turned out to be highly inefficient with the limiting step being metabolite transfer from *E. coli* to the yeast cells. An additional alternative approach to simultaneously transiently express all putative pathway genes in *Nicotiana benthamiana* (a close relative to tobacco) was also attempted, but was unsuccessful because of endogenous glucosyltransferase activity in the host plant, which caused glucosylation of the intermediates formed, thus preventing them from functioning as substrates for subsequent reactions. These circumstances blocked our planned heterologous production of *cisoid*‐diterpenoids in yeast and tobacco, and also prevented us from using this system for the functional characterization of P450 candidate genes. These experimental setbacks prevented us from establishing small‐scale production in yeast of *cisoid*‐diterpenoids with interesting medical properties, which could have formed the basis for patenting.

Although we were unable to develop a production system for *Eremophila* diterpenoid biosynthesis during this project, the identification of the initial biosynthetic steps towards three major diterpene backbones, as well as the identification of candidate genes for the cytochrome P450 enzymes catalyzing further oxygenation reactions, provide a guide for the elucidation of the full biosynthetic routes. Likewise, the enzyme systems are now available to provide scaffolds for further modification and bioactivity testing. This will open up the possibility of biotechnological production (Figures 3 and 5) (Gericke et al., 2020).

The overall aim of our research initiative was to access the full chemical space of the genus *Eremophila* and allied genera. Based on detailed HPLC‐MS analyses of 80% of the species in tribe Myoporeae, the metabolite diversity was analyzed in a chemo‐evolutionary framework combining molecular phylogenetics and state‐of‐the‐art computational metabolomics tools (Figures 3 and 5) (Gericke et al., 2021). Clear phylogenetic patterns with closely related species having similar chemical profiles were revealed when metabolic clusters and phylogenetic analyses were combined in the form of tanglegrams (Figure 3) (Gericke et al., 2020). The chemo‐evolutionary relationships were placed in a systematic context by integrating information from other researchers about leaf morphology (hairy, glabrous and resinous or non‐resinous leaves; Chinnock, 2007), environmental factors and geographical distribution (arid and semi‐arid or coastal regions; Figure 2; Gericke et al., 2021), traditional medicinal uses recorded in the literature (Barr et al., 1993; Latz, 1995; Richmond, 1993; Richmond & Ghisalberti, 1995) and antibacterial effects (Liu et al., 2006; Lyddiard & Greatrex, 2018; Ndi et al., 2007) to augment our understanding of complex interactions in biological systems (Figures 3, 4, 5, 6).

To get a different overview of the metabolome of the tribe Myoporeae, chemical subnetworks, each representing a chemical family of structurally related chemical structures, were built using the accurate molecular masses and the fragmentation patterns provided by the recorded mass spectra (Figure 4). The validity of the predicted structures assigned to subnetworks was greatly improved by including numerous reference compounds isolated from *Eremophila* species in the analyses. Among the reference compounds were a large number of flavonoids as well as sesquiterpenoids and diterpenoids, each of which was anchored within a specific network (Gericke et al., 2020, 2021). The results of these subnetwork analyses can in future be used to predict the type of enzymes involved in the respective chemical conversions and thereby guide the elucidation of the biosynthetic routes to chemicals of interest (Figures 4, 5, 6) (Gericke et al., 2021).

### Ethical dilemmas encountered in the project regarding benefit sharing with Australia's First Peoples

As stated in the introduction, it is important to recognize that many *Eremophila* species hold immense cultural significance to Australia's First Peoples. Already prior to obtaining the Novo Nordisk Foundation Interdisciplinary Synergy grant, Susan Semple worked collaboratively with some Aboriginal Corporations. Our joint efforts to integrate such collaborations in our current project were guided by Susan Semple's experiences.

As natural science researchers, we are obligated to follow the national laws of the country from which we obtain biological resources for research purposes. The biological resources include genetic and biochemical resources and the biochemical composition of genetic materials. In Australia, there is a nationally agreed upon approach for the access and use of biological resources. However, each state and territory government are responsible for managing this access under its own laws (Australian Government, Department of Agriculture, Water and the Environment, 2021a). In the state of Western Australia, where the research efforts of this project were focused (Figure 2), state legislation requires a license to collect flora. One license applies for taking flora for ‘scientific purposes’, where no foreseeable commercial outcome is envisioned. This is the type of license under which the initial work on the DNA phylogeny of *Eremophila* was undertaken (Fowler et al., 2020, 2021). A similar license is needed from other state governments in Australia. A second type of license is based on a bioprospecting agreement and is required in cases where flora is taken and a commercial outcome is foreseeable. This was the type of license obtained to permit us to carry out the polypharmacological profiling analyses of the *Eremophila* genus (Gericke et al., 2020; Kjaerulff et al., 2020; Pedersen et al., 2020; Petersen et al., 2019; Zhao et al., 2018; Zhao, Kjaerulff, et al., 2019). This license, negotiated with the Western Australian government agency, allows for the collection of samples from specified genera and/or species for a fee as well as for some longer term monetary and non‐monetary obligations.

The Nagoya Protocol on Access to Genetic Resources and the Fair and Equitable Sharing of Benefits Arising from their Utilization to the Convention on Biological Diversity (also known as the Nagoya Protocol on Access and Benefit Sharing) (Secretariat of the Convention on Biological Diversity, 2011) is an International Agreement that aims to ensure that the benefits from the use of genetic resources such as plant materials are shared in a fair and equitable way. This includes sharing with the country of origin and with Indigenous Peoples and local communities who hold these resources or knowledge associated with them. Australia ratified the Convention on Biological Diversity on 18 June 1993, and although Australia is a signatory to the Nagoya Protocol, it has not yet ratified it. The Australian federal government states that Australia's existing domestic measures are consistent with the Nagoya Protocol (Australian Government, Department of Agriculture, Water and the Environment, 2021b). The responsibility for biodiversity conservation and access and benefit sharing, including protecting the rights of Australian Indigenous Peoples and their knowledge, is distributed across different states and territories as well as the federal government. *Eremophila* species hold immense cultural significance to Australia's First Peoples. Knowing this and recognizing that the conditions for obtaining bioprospecting agreements are rapidly changing into more restrictive formats, the project team wanted to go beyond the minimum legal required standard for benefit sharing.

As a team of natural scientists wishing to study *Eremophila* and allied genera, we were faced with negotiating our way through the state and national/international legislation so that we could carry out our proposed research in a legitimate manner. The process at the level of the Government of Western Australia was facilitated by face‐to‐face meetings in Perth with the senior manager responsible at the Department of Parks and Wildlife, and progressed well as processes were in place and it was clear which government agency was needed to negotiate with to receive the appropriate license(s), based on specific agreed‐upon conditions. In our case, this included a way of sharing any future benefits, such as profits from the commercialization of plant compounds, with Aboriginal Peoples who could claim custodianship of the plants or knowledge associated with them, as stipulated in the Nagoya Protocol.

The bioprospecting agreement and the licenses obtained covered the legal governing of the research. To acknowledge the importance of many *Eremophila* species to Aboriginal Peoples, we faced several challenges, most of which were related to our desire to investigate the presence of bioactive natural products and chemo‐evolutionary relationships across the entire *Eremophila* genus, instead of in a single or a few species. Accordingly, processes to identify the appropriate Aboriginal Custodians of each *Eremophila* species were not available. Records of accumulated knowledge within the many different groupings of First Nations Peoples of Australia associated with each of all these *Eremophila* species were also scarce. Some of the complexities that we faced were as follows:

• ** The Indigenous Peoples of Australia (the Aboriginal and Torres Strait Islander Peoples) are many different and distinct groups, with their own cultures, customs, languages and laws.** The different nations, language and social groups have been illustrated in the Australian Institute of Aboriginal and Torres Strait Islander Studies (AIATSIS) map of Indigenous Australia (Australian Institute of Aboriginal and Torres Strait Islander Studies, 2022a). There are over 250 Indigenous languages in Australia with approximately 800 dialects (Australian Institute of Aboriginal and Torres Strait Islander Studies, 2022b), and with about 90 languages in Western Australia. Within those groups are other subgroupings that have ties to particular areas of the country. Aboriginal Peoples are the First Peoples of the Australian mainland, including Tasmania and some other islands. There is no centralized authority or Land Council that has the right to speak on behalf of all Aboriginal Peoples. As highlighted in the recently published Traditional Knowledge Guidelines around biodiscovery in the Australian state of Queensland (Queensland Government, 2021), custodians with the authority to speak for a particular area of the country may be identified through a variety of representative organizations.
• ** It is well known that several *Eremophila* species are culturally important plants for Australian Aboriginal Peoples.** *Eremophila* species have been and continue to be used in cultural ceremonies and practices and for medicinal purposes. The published records of Aboriginal Peoples’ use of *Eremophila* species (Smith, 1991; Barr et al., 1993; Richmond, 1993; Ghisalberti, 1994; Latz, 1995) have recorded about 25 species with medicinal uses, but most of these records come from Central Australia not Western Australia, where most of the plants in our present study were collected. Consequently, there is incomplete publicly available knowledge and records on which *Eremophila* species have been and continue to be used. Sharing particular traditional knowledge in the public domain may not be culturally appropriate. Further, sharing knowledge publicly may be problematic for First Nations communities where mechanisms to protect this knowledge are currently limited (Janke & Sentina, 2018).
• ***Eremophila* is a large genus of plants.** We wished to collect and analyze most of the *Eremophila* genus in Western Australia, which involved approximately 270 species and subspecies plus a number of undescribed taxa (Figure 3). Therefore, this would involve collecting specimens over an extremely large area, covering current crown (Government) land and the country of many different Aboriginal groups.
• ** The geographical distribution of the individual *Eremophila* species vary considerably.** Species distributions range from local endemics, i.e. those confined to a single hill or small‐scale locality, to those distributed across multiple states or much of the continent.
• ** Uses of and relationships with *Eremophila* species vary between Aboriginal groups.** Where a species of *Eremophila* has a wide distribution and grows in country belonging to multiple different groups of Aboriginal Peoples, not all groups may use or have recorded the use of that *Eremophila* species. Where a language group has recorded the use of a species of *Eremophila* there may be subgroups or families who only harvest specific plants from specific locations, or use plants in a specific way. Some of these local plants may have been cultivated, developed or selected over time through thousands of years of traditional land management practices (Fletcher et al., 2021; Gammage, 2011), and so may differ in their properties compared with the same species in other parts of the geographical distribution. This has been termed ‘embedded’ or ‘embodied’ traditional knowledge (International Indigenous Forum on Biodiversity,^2021^).
• ***Eremophila* chemistry is complex.** As chemical analysis of *Eremophila* species has shown (Gericke et al., 2021), some bioactive compounds may be found in several different *Eremophila* species distributed in different areas of the country. A single species may also be variable in the presence or absence of a particular compound in different locations of the country (Gericke et al., 2021). It may therefore be difficult to link an individual bioactive compound to a particular species or an area of the country in which it is collected.

Collaborative partnerships sharing Aboriginal and Western scientific perspectives in the investigation of Australian plants have been previously documented based on collaborative relationships between a single or small number of Aboriginal groups and science researchers from a single or a few institutions. These have also looked at selected species rather than a whole genus (Jamie, 2021; Janke, 2018). As outlined above, our desire to work across the entire *Eremophila* genus presented a number of issues and dilemmas. We approached these in a way that complied with the obligations under current Australian law and the Nagoya Protocol and met our desire to go beyond the minimum legal standard, to acknowledge the importance of many *Eremophila* species to Aboriginal Peoples. We took the following steps:

•  Although only a small number of the *Eremophila* species had been recorded as being specifically used by Australian Aboriginal Peoples, we took the view that all *Eremophila* species were included under any ownership claim. We made sure that we documented our activities on all species that we collected, assuming that any or all could be claimed in the future.
• While recognizing the complexity generated when working on a large number of species collected across country belonging to a large number of different groups of Aboriginal Peoples, we endeavored to set up a ‘trust fund’ that would be available to support locally initiated and driven projects for any Aboriginal group that claimed ownership of *Eremophila* species, or the knowledge associated with it.

Several indications for commercialization opportunities have arisen in the course of our investigations of *Eremophila*, but are far from having matured during the 3‐year project period. As the normal time frame for commercialization is 10–15 years, this is no surprise. Nevertheless, we hope that our cross‐disciplinary efforts and the resulting scientific publications open the door for continued future economic support from non‐profit foundations and foster industrial collaboration agreements and possibly down payments. To make this of benefit to Aboriginal Peoples, we had negotiated acceptance from the University of Copenhagen that a significant percentage of down payments and royalties derived from collaboration agreements or patenting would be donated to the trust fund. Up until now, we have unfortunately not been able to acquire such additional income, but we are still trying and eager to meet the initially stipulated goals of bringing back economic benefits to Aboriginal groups and communities.

We initiated work to establish the trust fund structure at the start of the 3‐year project period. This was to ensure that any future benefits arising from the research would be distributed in accordance with the bioprospecting agreement. A challenge with this is implementing a structure for such a trust fund that can deal with the fact that commercial outcomes and hence funds from research may not be gained until many years after the initial discovery. Going forward, the trust fund structure will be further developed in accordance with the requirements of the Government of Western Australia. Work with an existing trust fund already working to the benefit of Australia's First Peoples would save years of lawyer fees that would be unaffordable unless extra funds were provided.

Remembering that there was no clear guidance on how to proceed, as well as no registration of intellectual property, we undertook this bioprospecting project in good faith, conforming with legislation at the time, and tried to instigate a solution to ownership issues of benefit sharing as stipulated in the Nagoya Protocol. The project team still possesses the samples we collected, which we are willing to share with the rest of the research community, but only under an agreement that will conform to our bioprospecting agreement with the Western Australian Government and with the spirit of the Nagoya Protocol.

Digital sequence and metabolic data associated with the published phylogenetic and chemical network components of this research have been made publicly accessible in online data repositories following standard publication requirements (Gericke et al., 2021). It was our intention that this work forms a foundation of knowledge upon which further research can be based. However, this raises several issues around data use and benefit sharing. Although the Nagoya Protocol currently governs the use of physically collected samples such as plant material and associated traditional knowledge, there is currently continuing international discussion about whether digital sequence information (such as RNA, DNA and amino acid sequences; Watanabe, 2019) arising from physical samples (such as the sequence information obtained in this study) should be similarly governed (Karger & Scholz, 2021, Ambler et al., 2021, International Indigenous Forum on Biodiversity, 2021). As researchers, we are still grappling with how results are best communicated and shared whilst upholding the values of the Nagoya Protocol, particularly with regards to upholding benefit sharing when third parties can utilize the metabolic and genetic sequence data generated. In the case of our study, the vast number of DNA sequences made publicly available are those for chloroplast genomes and nuclear ribosomal DNA arrays that are unrelated to the natural products and bioassays we have described, i.e. sequences that are not directly relevant to potential pharmaceutical development or synthesis from *Eremophila*. However, we do disclose the sequences of unique *cis*‐prenyltransferases catalyzing the formation of nerylneryl diphosphate and of diterpene synthases catalyzing the formation of the serrulatane, viscidane and cembrane backbones giving rise to the wide range of structurally complex diterpenoids present in the *Eremophila* genus (Gericke et al., 2020). The genes encoding these *cis‐*prenyltransferases and diterpene synthases are going to play central roles in the bioproduction of diterpenoids with pharmaceutical potential.

We hope that the issues and limitations raised here can add to conversations among stakeholders, including First Nations Peoples and organizations, the plant sciences research community, lawyers and governments, examining how regulatory systems can be extended to improve benefit sharing in large studies including entire plant families or genera that include species used by First Nations Peoples. In this context, and as outlined in the original proposal to the Novo Nordisk Foundation, we took the opportunity to organize the ‘First Cross‐Continental *Eremophila* Conference’ and invite researchers working with Eremophilas, including representatives from a few Aboriginal communities. The conference took place in Melbourne on 28–30 October 2019 (https://synbio.ku.dk/calendar/2019/1st‐cross‐continent‐eremophila‐conference/conference‐report/). Thanks to extensive prior communications from the organizing committee involving both Australians and Danes to establish the foundation for a fruitful meeting, the conference provided space for a multi‐stakeholder sensitive dialog on cultural differences, ownership of the land and plants, and future perspectives. Strong statements and subsequent discussions were essential for helping participants understand the ethical and scientific issues from different perspectives (see Supplementary Materials. Suplementary Box 1 outlining the meeting schedule and discussion summary).

**Figure 7:**
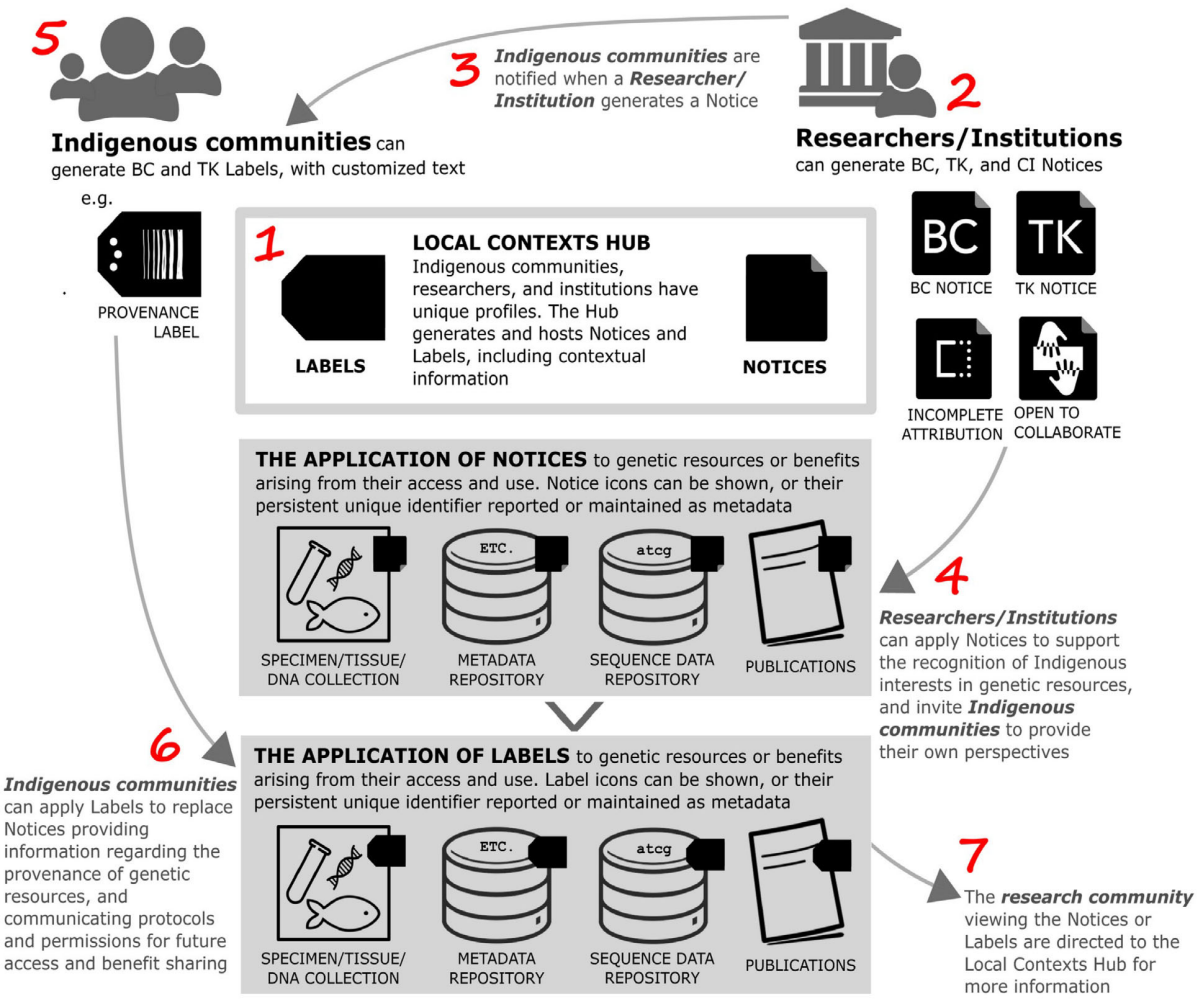
Creating space for Indigenous perspectives on access and benefit sharing. Schematic illustration to encourage researcher and institutional use of the Local Contexts Notices: BC, Biocultural; TK, Traditional Knowledge; CI, Cultural Institution. Figure provided by Liggins et al. (*Molecular Ecology* 2021;30:2477–2482). [Colour figure can be viewed at wileyonlinelibrary.com]

After we signed the bioprospecting agreement in 2017, a number of proactive bottom‐up initiatives have been taken to invigorate the Nagoya Protocol and the principle of benefit sharing by creating space for Indigenous communities to provide their perspectives, including their own definitions and aspirations associated with access and benefit sharing (Figure 7) (Marden et al., 2021; Local Context Team, 2022). We recognize that this in no way forces researchers and industries to engage in benefit sharing. However, the UN Sustainable Developmental Goal to reduce inequality and new bottom‐up initiatives to introduce Biocultural, Traditional Knowledge and Cultural Institution Notices serves to signal Indigenous provenance of the genetic resources used and rights of Indigenous Peoples to define their future uses (Liggins et al., 2021).

## KEY EXPERIENCES AND CHALLENGES

**Figure 8:**
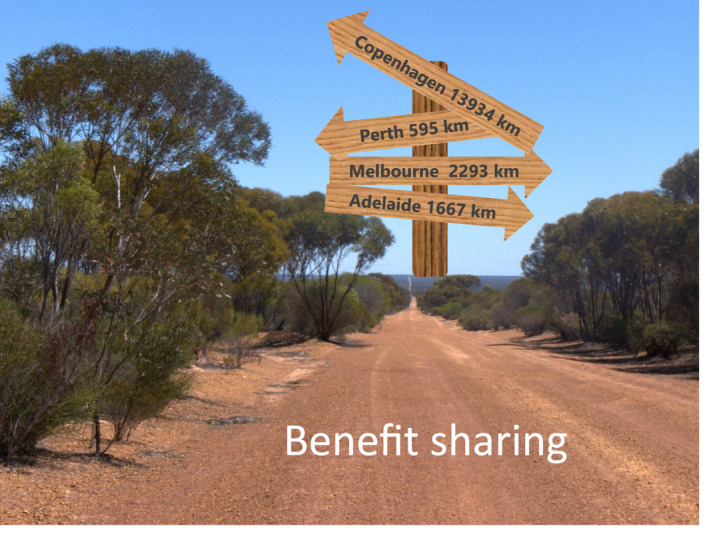
The landscapes of natural science and ethical dilemmas. The cross‐continental and highly interdisciplinary research initiative was based on a cross‐continental close collaboration between researchers in Australia and Denmark, with the aim to establish an atlas of the bioactive natural products present in the plant genus *Eremophila*. The road map available to properly address the ethical dilemmas encountered in the project regarding benefit sharing with Australia's First Peoples provided non‐coherent guidance on how to navigate this issue. [Colour figure can be viewed at wileyonlinelibrary.com]

In natural science, a key criterion for success and continued funding is to publish the results obtained in high‐impact journals. This parameter significantly affects our opportunities for continued funding. As outlined above, such criteria pose serious dilemmas and potential conflicts when the research carried out is highly cross‐disciplinary (Figure 8). In direct relation to this project funded by the Novo Nordisk Foundation Interdisciplinary Synergy program, we have published 12 scientific papers on the phylogeny of *Eremophila* and the presence of bioactive natural products and their biosynthesis, and more papers are in the pipeline.

As outlined above, all researchers involved in the 3‐year research initiative ‘Desert‐Loving Therapeutics’ funded with 2.0 million Euro by the Novo Nordisk Foundation Interdisciplinary Synergy program were successful in providing new knowledge on the Australian genus *Eremophila*. We managed to bring together researchers with the desired competences to advance such an interdisciplinary research project. Key success criteria included willingness and ability to collaborate, mutual trust and dedication towards fulfilling the aims. The result was the integration of a comprehensive phylogeny of *Eremophila* based on DNA sequencing with extensive chemical profiling data to provide a chemo‐evolutionary perspective on this large genus (Figure 3). The results of this analysis can and will be used to guide future research into the evolution of the unique phytochemistry of *Eremophila* species, and to pinpoint which species or groups of species should be the focus of future research on identifying new natural products.

At the biochemical level, we significantly extended the cognizance of the richness and interesting bioactivities of diterpenoids derived from serrulatane, viscidane and cembrane‐type scaffolds in *Eremophila* (Figures 3, 5, 6). No previous studies on the biosynthesis of diterpenoids in *Eremophila* were reported. We were successful in identifying the enzymes responsible for the biosynthesis of these scaffolds and were surprised to discover *cis*‐prenyl‐diphosphate‐based diterpenoid metabolism in this genus.

Metabolite profiling of *E. oppositifolia* revealed that this species possessed the unique ability to produce monomeric as well as dimeric branched‐chain fatty acids. The occurrence of branched‐chain fatty acids in plants is rare and the presence of dimers of these have not previously been reported in plants (Figure 6). The formation of the dimers may be envisioned to be catalyzed by Diels–Alder cycloaddition reactions, but time constraints prevented us from addressing this experimentally.

A final goal stipulated in our application to the Novo Nordisk Foundation Interdisciplinary Synergy Program was to establish heterologous production systems for some of the natural products isolated that showed interesting bioactivities. We attempted to engineer production systems in yeast but were unable to functionally express the *cis*‐prenyltransferases that catalyze the formation of the *cisoid*‐diterpenoid precursor, nerylneryl diphosphate. Attempts at overcoming this obstacle using yeast/*E. coli* co‐culturing systems were also unsuccessful because of the inefficient transfer of metabolites between host organisms. These experimental setbacks prevented us from producing any *cisoid*‐diterpenoids in yeast. Nevertheless, it was indeed a great experience and opportunity to carry out this interdisciplinary research on the unique set of *Eremophila* samples collected during the 3‐year project period. Throughout the project as well as continuing after the project period we are committed to ensure benefit sharing with the Aboriginal Peoples of Australia. With the extension of our bioprospecting agreement to 2027, we continue to abide by the legal framework outlined herein.

Our recent open‐access publication in *The Plant Journal* (Gericke et al., 2021) on navigating through chemical space and evolutionary time across the Australian continent in the plant genus *Eremophila* was published as a resource article because it provides an account of the huge data set of the natural products discovered and structurally characterized. This means that researchers from other universities and from industries now have access to the data and are free to move ahead using these data also with a mindset of commercialization.

In the spirit of our bioprospecting agreement and to adhere to our personal wishes of benefit sharing, we include the following statement at the end of this publication:

If you use the information provided here or from our other recent publications to make commercial products, we urge you to strongly consider the Nagoya Protocol requirements to share the benefit with the Aboriginal communities or groups in the areas where these plant species grow. We acknowledge that the work discussed here took place on the lands of Aboriginal Peoples who are the custodians of this land, and acknowledge and pay our respects to their elders, past and present.

## CONFLICT OF INTEREST

The authors declare that they have no conflicts of interest associated with this work.

## Supporting information


**Appendix S1** Supplementary material.
