# Supplementary material for: Biodiscoveries within the Australian plant genus Eremophila based on international and interdisciplinary collaboration: results and perspectives on outstanding ethical dilemmas
Source: Plant J. 2022 Jul 23;111(4):936–53. doi: 10.1111/tpj.15866 (PMC9543726; doi:10.1111/tpj.15866)
Supplement: Supplementary file 1 — Appendix S1 Supplementary material. [file TPJ-111-936-s001.docx]

**Supplementary material:**

First Cross-Continent Eremophila Conference – sharing knowledge and opening space for sensitive dialogues

To facilitate scientific knowledge-sharing and bring together as many aspects of *Eremophila* knowledge and ownership for mutual benefit, we organized the 1^st^ Cross-Continent Eremophila Conference. The conference took place on October 28-30^th^, 2019 at the premises of The University of Melbourne.

**We acknowledge that the conference took place on the lands of the Wurundjeri people who are the Traditional Custodians of this land, and acknowledge and pay our respects to their Elders past and present.**

**- Paying our respects to the Aboriginal people in the way that is becoming customary in Australia**

An exciting diversity of *Eremophila* experts participated in the conference: From plant scientists to neurobiologists and pharmacologists, from social scientists to social enterprise entrepreneurs, guerilla gardeners and private collectors. Together the participants brought together a rich and valuable variety of deep and passionate knowledge about the *Eremophilas*. Moreover, the conference participants managed to create a forum of dialogue that facilitated sensitive and difficult conversations of the multileveled ownership, cultural differences and connection to the Australian land, where Australia’s First Peoples– the Aboriginal Peoples- have lived for thousands of years whereas the Europeans arrived in Australia less than 250 years ago. It became clear that our conference with the participation of researchers from other continents was a fruitful forum.

**Scientific Highlights of the conference:**

**Section on Natural Science**

- [Birger Lindberg Møller](https://synbio.ku.dk/blm/) chaired the conference and introduced the ‘Desert-Loving Therapeutics’ project funded by Novo Nordisk Foundation, its focus on *Eremophilas* and the larger implications.
- [Bob Chinnock](https://www.environment.sa.gov.au/Knowledge_Bank/Science_and_research/State_Herbarium/Our_people/Dr_RJ_Bob_Chinnock) presented his foundational work on *Eremophila* as documented in his monograph on their taxonomy and phylogeny.
- [Bevan Buirchell](https://www.linkedin.com/in/bevan-buirchell-932b3520/?originalSubdomain=au)shared his extensive botanical knowledge of the *Eremophila* and his deep knowledge of their growth habitats in the Western Australian desert enabling him to sample species at the most remote places.
- [Mike Bayly](https://plantsystematicsblog.wordpress.com/mike-bayly/) and [**Rach**](https://plantsystematicsblog.wordpress.com/current-students/)**ael Fowler** from Melbourne University presented their phylogenetic work on *Eremophila* and tribe Myoporeae.
- [Oliver Gericke](https://plen.ku.dk/english/employees/?pure=en/persons/oliver-gericke(e5cc7c13-1b80-41e4-8f32-a639cdccadea).html) from University of Copenhagen presented his work on molecular networking and chemo-phylogenetic relationships of *Eremophila* species, complementary to Rachael Fowlers work.
- [Susan Semple](https://people.unisa.edu.au/Susan.Semple%252520) and [Chi Ndi](https://people.unisa.edu.au/Chi.Ndi)from University of South Australia presented their analytical work on isolation of natural products from *Eremophila* species with focus on compounds inhibiting efflux pumps and thereby may be used to overcome multidrug resistance.
- [Dan Stærk](https://drug.ku.dk/staff/?pure=en/persons/321761) from the University of Copenhagen reported on the isolation and structural characterization of complex diterpenoids and dimeric branched fatty acids and on high-resolution bioactivity profiling of *Eremophila* extracts
- [Birger Lindberg Møller](https://synbio.ku.dk/blm/) from the University of Copenhagen presented on elucidation of the biosynthesis of core skeletons of complex diterpenoids and the production of these in heterologous hosts.
- [Claus Juul Løland](https://in.ku.dk/testarea/research/claus-loeland/) from the University of Copenhagen presented on the neuropharmacological effect of dimeric branched fatty acids on the dopamine transporters and potential pharmaceutical uses of the compounds.
- [Rohan Davis](https://au.linkedin.com/in/rohan-davis-33a713102) from Griffith University in Queensland presented his work with the establishment of the [NatureBank](https://www.griffith.edu.au/institute-drug-discovery/unique-resources/naturebank) and the inclusion of the Nagoya protocol in his research on indigenous resources.
- [Dan Murphy](https://www.rbg.vic.gov.au/staff/daniel-murphy/daniel-murph) and [Mike Whitehead](https://michaelwhitehead.net/curriculum-vitae/) from University of Melbourne presented data on the biogeography of *Eremophila* species and their interaction with pollinators.
- Bevan Buirchell presented data on his observed interactions between different *Eremophila* plants and insects and the implication for *Eremophila* phylogeny, showcasing his collaborative work with Gerry Cassis.
- The Eremophila Study Group is part of the Australian Native Plants Society of Australia ([ANPSA](http://www.anpsa.org.au/eremophilaSG/index.html)) and was represented by highly passionate and very keen participants and contributed with their broad spectrum of knowledge throughout the conference. Their knowledge, know-how and enormous variety of *Eremophilas* cultivated in their gardens represents a great and important resource also to the Novo Nordisk Fonden project

**Section on Biodiscovery and Benefit-Sharing**
This important conversation was in focus in session 2.

- [Susan Semple](https://people.unisa.edu.au/Susan.Semple%252520) from the University of South Australia presented her collaboration with Aboriginal custodian David Claudie from the Kuuku I’yu homelands in Cape York. Though he could not be present at the conference, the presentation had been prepared with him. He and the Chuulangun Aboriginal Corporation have been collaborating with Susan Semple for a number of years. Together they have explored the compounds of their traditional medicines found in the plants on homelands area.
- [Margaret Raven](https://www.arts.unsw.edu.au/our-people/margaret-raven) and [Daniel Robinson](https://www.arts.unsw.edu.au/our-people/daniel-robinson) are Social Scientists and presented their work and deep knowledge on access and benefit-sharing, customary law and culturally significant species, such as the *Eremophilas*. Their work is also focused on the implementation of the Nagoya protocol in Australia, where the agreement has been signed, but is not yet ratified. This is contributing to and creating challenges for the unfolding of new and respectful approaches to the Custodians of the land.
- [Peta Cobourn](https://www.bushbalm.com.au/) is the Social Enterprise Manager at the Purple House, a**n innovative Indigenous-owned and run health service based** in Alice Springs in Central Australia and presented the history behind the establishment of the Purple House’s life-saving work of bringing dialysis to remote communities and making Purple House a ‘safe-space’ for indigenous people. The initiative has founded the corporative Bush Balm that produces lotions and balms made with ingredients from *Eremophilas*. The plants used in the products are collected by their Traditional Custodians around Alice Springs and prepared according to their tradition and knowledge.
- [Joanne Jamie](https://researchers.mq.edu.au/en/persons/joanne-jamie) is a Natural Product Chemist and shared with us her journey of involving the Yaegl Elders and the young generations of the Aboriginal people around Macquarie University in science and traditional knowledge initiatives. This kind of empowerment has had an immense influence on the younger generation of First Australians.
- [Andrew Gleadow](https://findanexpert.unimelb.edu.au/profile/16040-andy-gleadow) from the University of Melbourne is the Chief Science Advisor to Rock Art Australia (previously Kimberley Foundation) and presented his and the foundation’s work in finding and dating rock art in the Australian Kimberleys. This work, which involves close collaboration and negotiation with local communities has been highly rewarding, not only science-wise, but also personally for Andrew Gleadow.
- [Colette Geier](https://www.linkedin.com/in/colette-geier-3b70ba77/?originalSubdomain=au) recently begun her PhD studies on the effect of Ngurambang (country and customary practice) on the phytochemistry of Yadhandha (*Eremophila longifolia*). In her studies, she is exploring the effect of habitat, harvest and herbal preparation on the bioactive compounds present in the plant.

The two sessions laid the ground for a multi-facetted and at some instances aggravated dialogue on the presented research on the *Eremophila* and how to initiate a respectful accommodating dialogue and collaboration with Aboriginal communities taking into account benefit-sharing. As Margaret Raven stated **“This is not an easy conversation, but it is a necessary one”**. Many lives and ways of life are negatively affected by our lack of ability to find a fruitful ground for such collaborations move forward on.
